# Supplementary material for: Sources and upstream pathways of the densest overflow water in the Nordic Seas
Source: Nat Commun. 2020 Oct 23;11:5389. doi: 10.1038/s41467-020-19050-y (PMC7584654; doi:10.1038/s41467-020-19050-y)
Supplement: Supplementary file 1 — Supplementary information [file 41467_2020_19050_MOESM1_ESM.pdf]

Supplementary Information

Sources and upstream pathways of the densest overflow water in the Nordic Seas

Jie Huang[1,2,*], Robert S. Pickart[2], Rui Xin Huang[2], Peigen Lin[2], Ailin Brakstad[3], and Fanghua Xu[1]

[1]Ministry of Education Key Laboratory for Earth System Modeling, and Department of Earth System Science, Tsinghua University, Beijing, China

[2]Woods Hole Oceanographic Institution, Woods Hole, USA

[3]Geophysical Institute, University of Bergen, and Bjerknes Centre for Climate Research, Bergen, Norway

*Corresponding author: huangj15@mails.tsinghua.edu.cn

Supplementary Table 1 | Data sources with corresponding time periods and references.

**Data source**	**Years**	**Reference**
UDASH (Unified Database for Arctic and Subarctic Hydrography)	1980-2015	*Behrendt et al.,* (2018) https://doi.pangaea.de/10.1594/PANGAEA.872931
ICES (International Council for the Exploration of the Seas)	1980-2015	Data web page: http://ocean.ices.dk/HydChem/HydChem.aspx
WOD (World Ocean Database)	1980-2015	Data web page: www.noaa.gov/cgi-bin/OS5/SELECT/builder.pl
Argo float program	2001-2015	Data web page: https://doi.org/10.17882/42182
NISE (Norwegian Iceland Seas Experiment database)	1980-2009	*Nilsen et al.,* (2008), The NISE dataset, Faroese Fisheries Laboratory Tech. Rep. 08-01, 20pp
MFRI (Marine Freshwater and Research Institute of Iceland)	1980-2015	https://sjora.hafro.is
GLODAPv2 (Global Ocean Data Analysis Project version 2) - 2019	1980-2013	*Olsen et al.,* (2019) https://doi.org/10.5194/essd-11-1437-2019 https://doi.org/10.25921/xnme-wr20

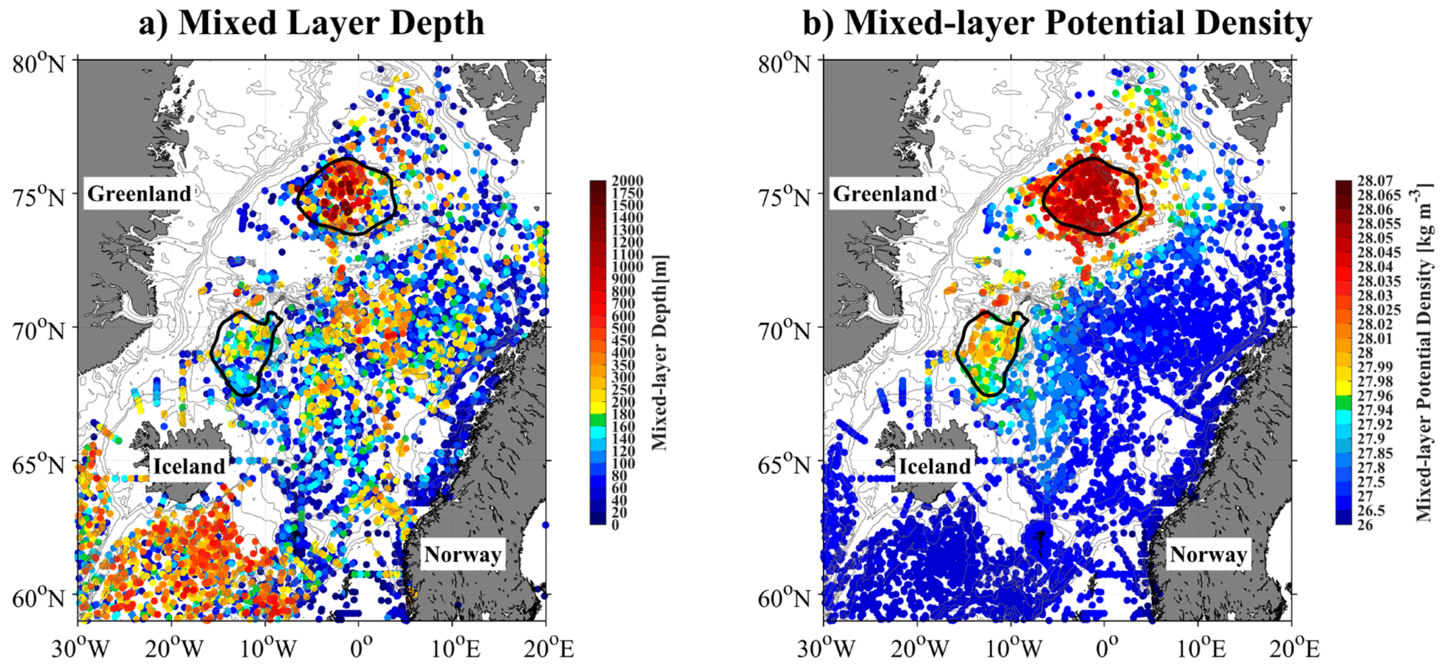

Supplementary Figure 1| Late-winter mixed-layer properties.

(a) Mixed-layer depth [m] from February-April. **(b)** Mixed-layer potential density [kg m^{-3}] from February-April. The Greenland Sea and Iceland Sea gyres are outlined by thick black contours (see caption to Fig. 2). The thin grey contours show the bathymetry from ETOPO2.

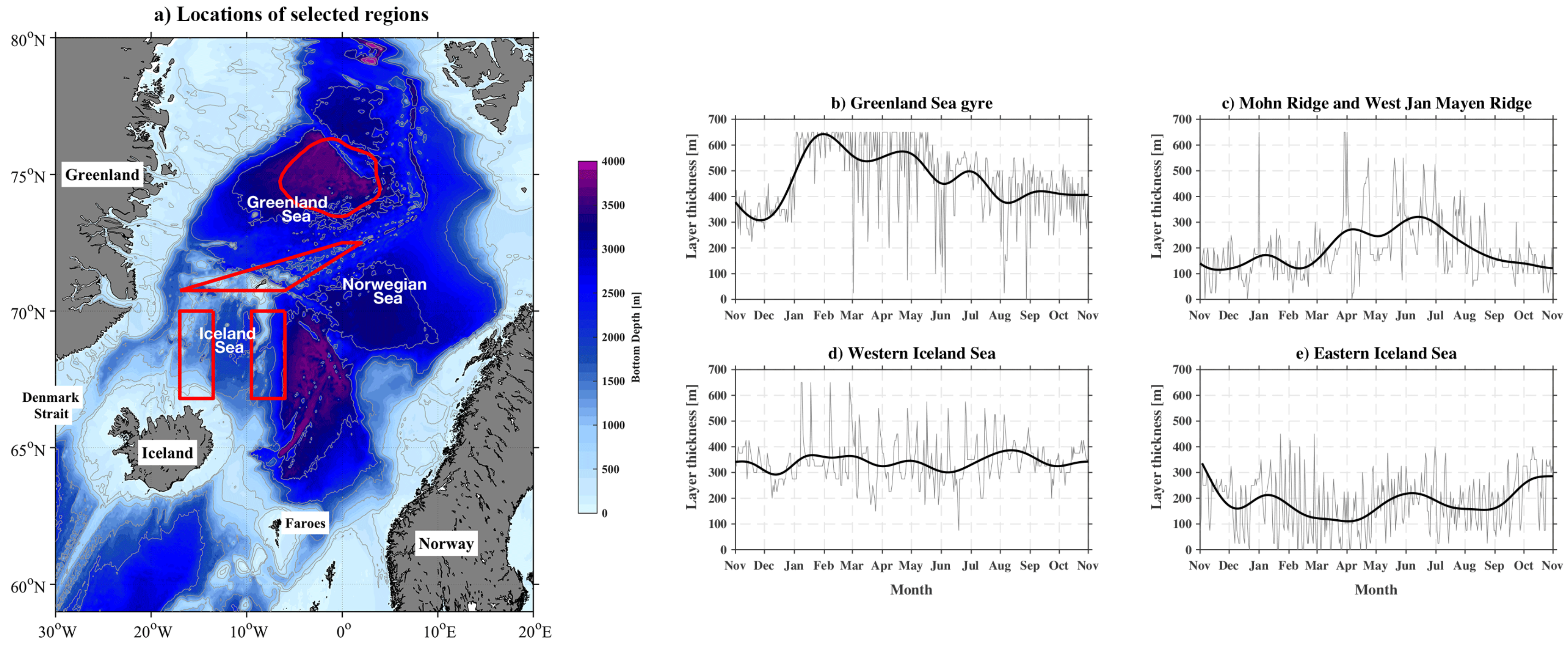

Supplementary Figure 2| Seasonal evolution of the layer thickness of small σ_0-π_0 distance in the upper 650 m of the water column, for the four regions outlined in red in (a), using data from 2005-15. (b) Greenland Sea gyre; (c) Mohn Ridge and West Jan Mayen Ridge; (d) Western Iceland Sea; and (e) Eastern Iceland Sea. The thin grey lines and thick black lines are daily and 60-day low passed time series averaged in each region, respectively.

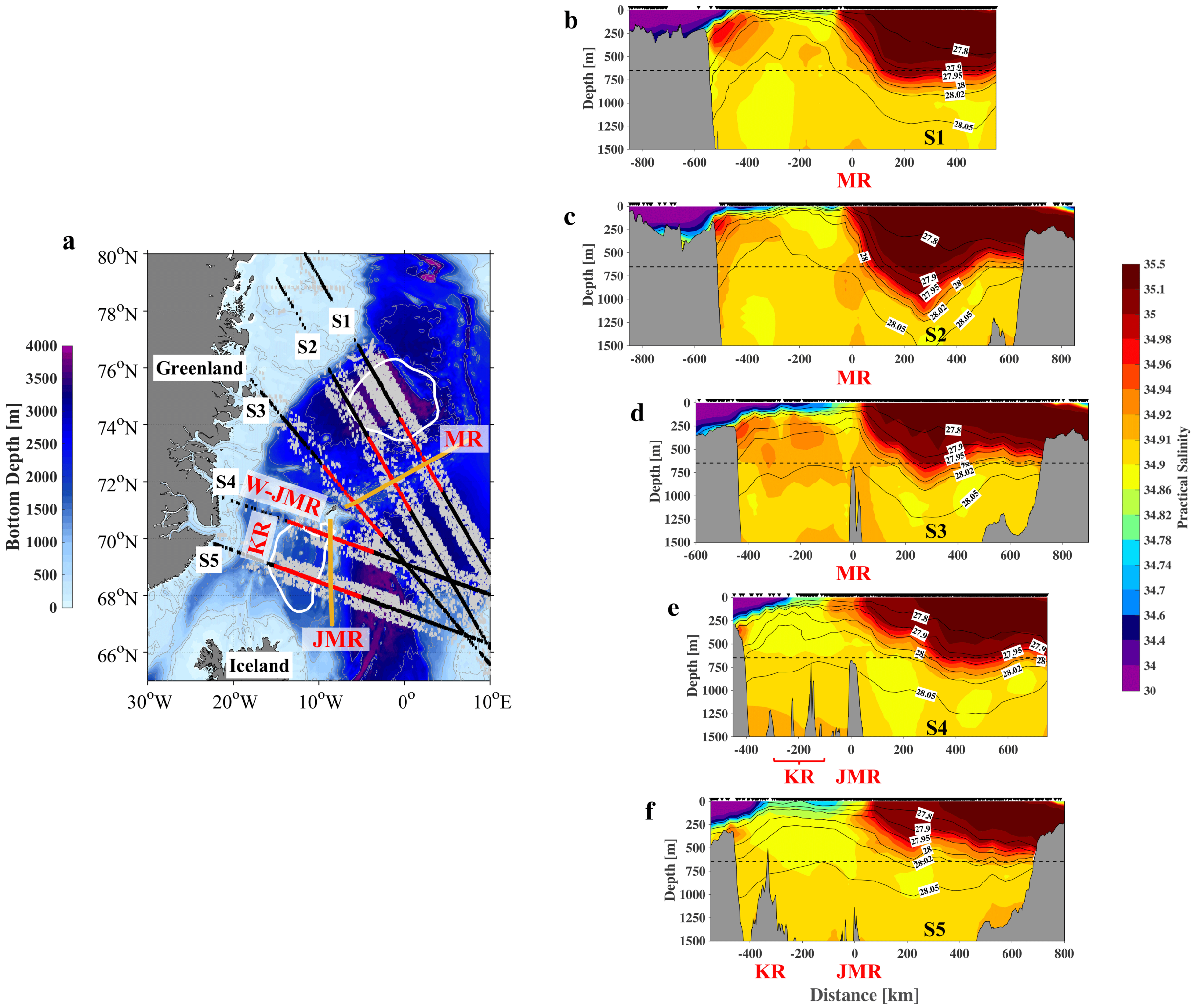

Supplementary Figure 3| Same as Figure 3 except for salinity.

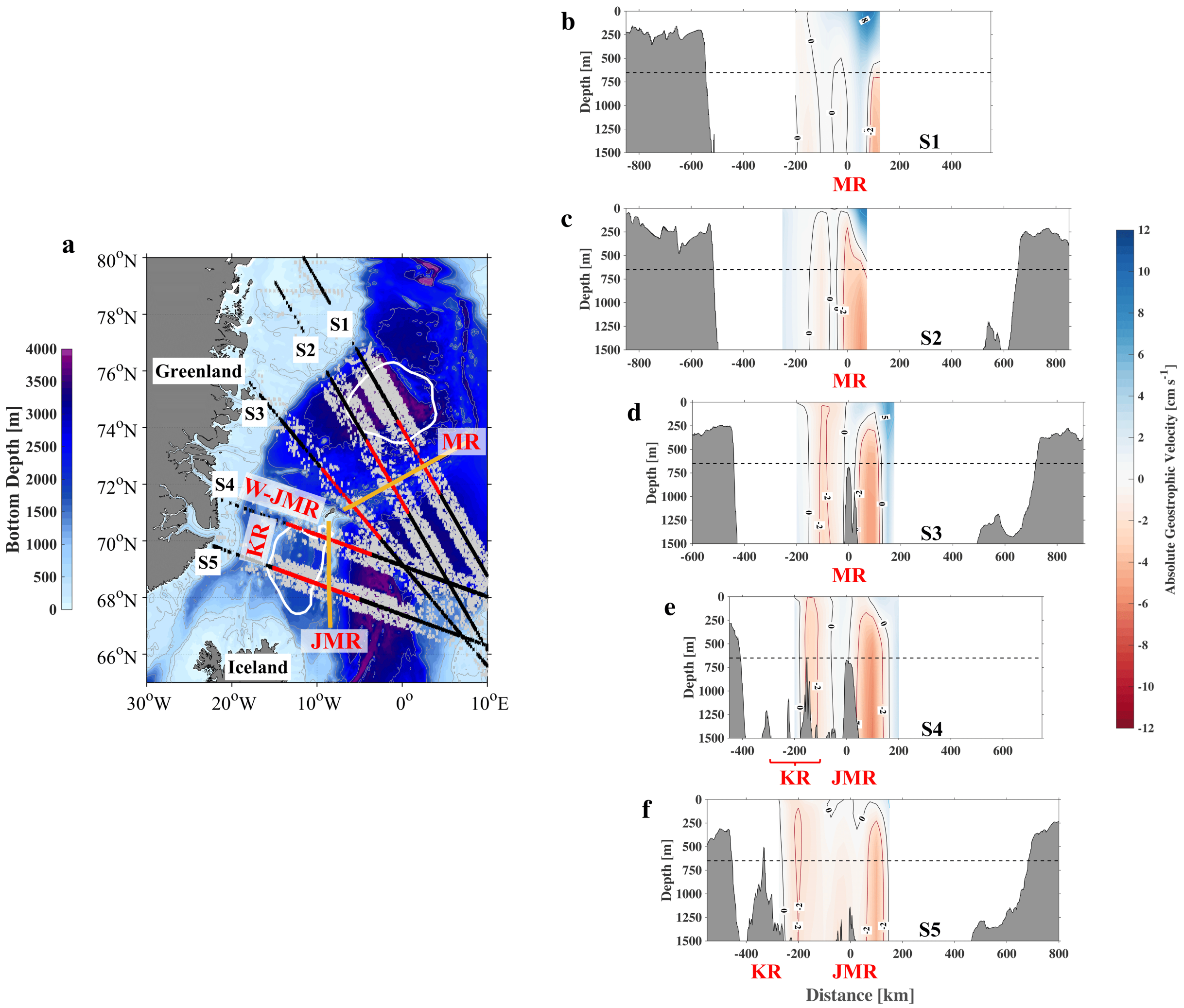

Supplementary Figure 4| Same as Figure 3 except for absolute geostrophic velocity. Negative velocities are equatorward.
